# Parecoxib Reduced Postsurgical Pain and Facilitated Movement More Than Patient Controlled Analgesia

**DOI:** 10.3389/fsurg.2022.799795

**Published:** 2022-04-06

**Authors:** Szu-Ching Chiu, Hanoch Livneh, Jin-Cheng Chen, Chia-Ming Chang, Honda Hsu, Tsay-I Chiang, Tzung-Yi Tsai

**Affiliations:** ^1^Department of Nursing, Dalin Tzuchi Hospital, The Buddhist Tzuchi Medical Foundation, Chiayi, Taiwan; ^2^Rehabilitation Counseling Program, Portland State University, Portland, OR, United States; ^3^Department of Neurosurgery, Dalin Tzuchi Hospital, The Buddhist Tzuchi Medical Foundation, Chiayi, Taiwan; ^4^School of Medicine, Tzu Chi University, Hualien, Taiwan; ^5^Department of Anesthesiology, Taichung Tzu Chi Hospital, The Buddhist Tzuchi Medical Foundation, Taichung, Taiwan; ^6^Division of Plastic Surgery, Dalin Tzuchi Hospital, The Buddhist Tzuchi Medical Foundation, Chiayi, Taiwan; ^7^Department of Nursing, Hungkuang University, Taichung, Taiwan; ^8^Department of Environmental and Occupational Health, College of Medicine, National Cheng Kung University, Tainan, Taiwan; ^9^Department of Medical Research, Dalin Tzuchi Hospital, The Buddhist Tzuchi Medical Foundation, Chiayi, Taiwan; ^10^Department of Nursing, Tzu Chi University of Science and Technology, Hualien, Taiwan

**Keywords:** patient-controlled analgesia, parecoxib, lumbar spinal fusion surgery, generalized estimating equations, pain

## Abstract

**Background:**

Postoperative pain management is an imperative issue for patients undergoing lumbar spinal fusion surgery. Delayed pain relief is associated with poor clinical outcomes. This study compared the effects of intravenously administered patient-controlled analgesia (PCA) with intravenous parecoxib, both commonly used methods for analgesic pain control after surgery.

**Methods:**

A non-randomized study was used to recruit 68 patients who were scheduled to receive lumbar spinal fusion surgery at a hospital in Taiwan from April through December of 2020. The group treated with parecoxib received an initial perioperative dose of parecoxib 40 mg during a 30-min period and then postoperative intravenous parecoxib at 40 mg per 12-h period, for 72 h. Those with PCA received morphine (0.4 mg/ml), droperidol (0.02 mg/ml), diphenhydramine (0.48 mg/ml), midazolam (0.02 mg/ml) and saline solution during the 3-day study course. Major outcomes, including visual scale pain score and Barthel index of activities of daily living, were collected via review of medical records at 4 times: 12, 24, 48 and 72 h after surgery. Comparative effects between two groups were assessed by the generalized estimating equations.

**Results:**

After adjusting for potential confounders, the administration of parecoxib was associated with a significant decrease in pain scores and an increase in the Barthel Index, when compared with the PCA group (all *p* < 0.05). Notably, both effects would maintain for 72 h after surgery.

**Discussion:**

This is the first trial of which the authors are aware, that supports intravenous parecoxib as significantly enhancing patient mobility, in addition to having pain control efficacy, when compared with PCA. This study could be used as a reference when instituting interventions to improve the adaptation process and clinical prognoses after lumbar spinal fusion surgery.

## Introduction

Pain is a frequent complication following surgery, especially for those receiving lumbar spinal fusion surgery. A recent comprehensive study, covering 2,996 cases undergoing lumbar surgery, reported that low-back pain was the most frequently reported presenting clinical symptom (73%), followed by radiculopathy (18%), lower-extremity weakness (8%), and bowel/bladder symptoms (0.3%) (1). The International Association for the Study of Pain (IASP) is the leading global organization supporting the practice of pain and pain relief, which further defines pain as an “unpleasant sensory and emotional experience associated with actual or potential tissue damage” (2). A recent study, however, indicated that nearly 60% of patients experienced inadequate pain control in the first 24 h after surgery (3). Once the individual undergoing operation did not experience adequate relief of post-operative pain, it is expected that the patient may experience delayed wound recovery and a higher stress response, thereby increasing length of hospital stay and healthcare costs (4, 5). Therefore, when managing cases of lumbar spinal fusion surgery, treating or lessening pain level is strongly indicated and is of high priority, in addition to following optimal management of all operating protocols.

Postoperative pain management is one of the most important issues throughout the perioperative period. Initially, patient-controlled analgesia (PCA), analgesics that are delivered immediately, upon patient demand, was utilized due to insufficient pain relief from conventional intramuscular delivery of opioids (6, 7). Morphine is the most studied and most commonly used intravenous drug for PCA. While it is the “first choice” for PCA, morphine may lead to adverse effects, such as nausea, vomiting, pruritus, respiratory depression, sedation, confusion, constipation, and urinary retention (8, 9).

As part of a significant increase in specialized diagnostic and therapeutic methods, the selective cyclooxygenase-2 (COX-2) inhibitor, parecoxib sodium (parecoxib), has been more recently introduced. The parecoxib treatment has been mainly employed for short-term treatment of postoperative pain, and also used for the treatment of perioperative analgesia to prevent or reduce severe postoperative acute pain (10). Currently, it is being widely utilized in a range of surgical procedures, such as gastrointestinal surgery (11), prostatectomy (12), gynecologic laparotomy surgery (6) and liver resection (7).

Based on the findings of studies conducted thus far, the authors found that reports of the effects of use of parecoxib on pain reduction were mixed, with several studies showing significant levels of efficacy, while others reporting no substantial benefit pain relief (11–13). Significantly, the previous studies did not adhere to the assumption of independence among participants' responses, and did not consider maturation effects caused by the intrinsic changes over time when evaluating intervention effects (11–13). These omissions may have led to premature or inaccurate conclusions. Faced with this gap in the literature, together with limited information regarding the head-to-head comparison between PCA and parecoxib among patients having undergone lumbar spinal fusion surgery, this study aimed to clarify the impact of parecoxib, as compared to PCA, among these patients, using a generalized estimating equations (GEEs) model.

## Methods

### Study Design and Participants

An observational, non-randomized study was designed to evaluate the effects of PCA in comparison with parecoxib among post-surgery patients at a hospital in Taiwan, from April through December of 2020. Just as the participants could freely choose the approach of preemptive analgesia upon their personal willingness, we utilized the observational, non-randomized approach to avoid disrupting or altering the pattern of present care. The inclusion criteria were: (i) being at least 20 years-old at the time of recruitment; (ii) having no cognitive impairment and severe complications; (iii) being able to communicate in either Mandarin or Taiwanese, and (14) having undergone lumbar spinal fusion surgery (14). To ensure participants' anonymity, all questionnaires were marked with an encryption code to facilitate data analysis, but showed no personal identifiers.

### Sample Size Calculation

The sample size needed for this repeated measures research design was estimated by using Cohen's methodology (15), where α was set to 0.05, power to 0.8, and the effect size to 0.16, focusing on the changes observed across four separate measurements of pain level in the two groups (16). It was determined, based on these chosen values, that a sample of at least 56 patients (each group including 28 patients) was required for data analysis (based on the G^*^POWER 3.1 analytical software, Franz Faul, Universitat Kiel, Germany).

### Intervention (Treatment)

All enrollees were allowed to choose between receiving either PCA or 40 mg parecoxib sodium intravenously twice a day for the following 3 days during the perioperative period. Those receiving parecoxib were initially given an intravenous bolus of 40 mg parecoxib, a parenteral COX-2-specific inhibitor, during the perioperation period. The subsequent 40 mg parecoxib was administered intravenously, at an even, computer-controlled, rate, every 12 h, throughout a period of 72 h. As to the participants of PCA group, they received the following medications: morphine (0.4 mg/ml); droperidol (0.02 mg/ml), diphenhydramine (0.48 mg/ml), midazolam (0.02 mg/ml) and saline solution, which was administered with a computerized intravenous infusion pump for 3 days. The pump intravenously infused a 3 ml bolus into patients when they pressed a button. Patients were instructed to press the button when experiencing postoperative pain. The lock-out time between each bolus injection was 5 mins. The maximum dose of PCA was limited to 50 ml every 4 h. While receiving the pain relief protocols, patients' vital signs (heart rate, respiratory rate, blood pressure and oxygen saturation) were monitored every 5 mins for the first hour and thereafter every 4 h. Supplemental oxygen by nasal cannula was provided once the patient's oxygen saturation fell below 95%. Besides, rescue analgesia was included in the protocol once the NRS was above seven, which indicated to severe postoperative pain and inadequate pain control.

### Outcome Assessments

Primary outcome indicators of this study comprised of levels of pain and mobility, as measured by a self-reported numerical rating scale (NRS) and the Barthel Index.

The NRS is a subjective measure in which individuals rate their pain on an eleven-point numerical scale. This scale extends from 0 (no pain at all) to 10 (worst imaginable pain), where higher scores are associated with a more severe degree of pain. The NRS was employed due to its simplicity, reproducibility, and sensitivity to relatively small changes in pain. A literature review by Safikhani and colleagues observed that the majority of published articles recommended the NRS as the most useful response scale for the assessment of pain in adults (17). Additionally, based on comparison with the Visual Analogue Scale (VAS), focusing on pain level as the gold standard, the NRS was determined to possess good concurrent validity, with a correlation coefficient of 0.92 (*p* < 0.001) (18).

Mobility was assessed using the Barthel Index, a widely used measure of the basic activities of daily living (ADL) (19). The Barthel Index includes 10 task items and is scored according to the amount of time or assistance required by the patient. The 10 items are: feeding; bathing; grooming; dressing; continence of bowels and bladder; transferring to and from a toilet; moving from wheelchair to bed and return; walking on a level surface for 45 meters, and; going up and down stairs. The total score ranges from 0 to 100, with lower scores representing greater nursing dependency. Lam et al. reported that the Barthel Index posseses sound predictive validity of clinical outcomes and its scores have shown good reliability, validity, and responsiveness among various clinical populations (20). The Barthel Index has demonstrated high inter-rater reliability (0.95) and test–retest reliability (0.89), as well as high correlations (0.74–0.8) with other measures of physical disability (19).

### Covariates

Additional items addressing patients' demographic and disease characteristics were developed according to clinical experience and literature review, and were gathered by a self-reported questionnaire. These data included gender, age, educational level, job status, monthly income and certain lifestyle factors, such as seeking other alternative treatments to reduce pain, as well as use of alcohol, recorded as “non-drinker” “current drinker” or “ex-drinker.” Baseline comorbid health conditions were self-reported and identified by a review of medical records, including hypertension, diabetes, hepatitis, peptic ulcer, and heart disease. Usage of analgesic medication prior to surgery was stratified into two categories, namely, (i) if one ever used relevant medications for a period of more than 3 months, or (ii) use them for <3 months, e.g., sulindac, celecoxib or tramadol.

### Data Collection Procedure

The study protocol was approved by the Research Ethics Committee of Dalin Tzu Chi Hospital (No. B10901022-1). At the study's initiation, researchers explained the purpose of study and its procedure to all participants. Signed informed consent was obtained after the patients understood and agreed to share their information with the research team. Thereafter, the authors applied an observer-blind approach for data collection. A trained interviewer, who was not familiar with participants or with the study design, was assigned to collect all information pertaining to the outcomes and covariates. All data were obtained at four time points after surgery, namely at 12, 24, 48, and 72 h. All participants were followed from the date of enrollment until the end of the follow-up and were given the option to withdraw from the study at any time without any penalty.

### Statistical Analysis

Descriptive and inferential statistical analyses were conducted in accordance with the study aims. Descriptive parameters, including means, standard deviations and percentages, were used to describe the distributions of demographic and disease data. The baseline differences between the two groups were compared using *t*-test and χ^2^ test as applicable. To evaluate the effect of treatment on the two groups, across the entire follow-up period, the generalized estimating equations (GEEs) procedure was utilized to assess the change pattern of NRS and Barthel Index. GEEs is a form of regression analysis which extends generalized linear models into a regression procedure with correlated observations within subjects. In a GEEs procedure, the standard errors of estimate are corrected for repeated measurement. In the present study, the baseline heterogeneity (differences existing before the intervention) between the experimental and control groups, and the maturation effects (changes in outcome variables resulting from the passage of time) were all controlled for by applying the GEEs method. All analyses were conducted using SAS statistical software, Version 9.3 together with SPSS 22.0. A *p*-value of <0.05 was considered statistically significant.

## Results

### Demographic and Disease Characteristics of Participants

A total of 68 patients who had lumbar spinal fusion surgery were enrolled in the study, with 36 in the PCA group and 32 in the parecoxib group. All 68 patients completed the follow-up material included in this study. Patients ranged in age from 40 to 76 years, with a mean of 60.6 years. Most patients were female (67.6%), employed (70.6%), and had low level of alcohol consumption (88.2%). The majority of patients had a monthly income of New Taiwan Dollars (NTD) ≤ 40,000 (52.9%) and reported having lower level of education (66.2%, defined as below 9th grade). In terms of disease characteristics, most of the enrolled patients presented without comorbidities and had ever been prescribed the anesthetic medications used in this study, prior to their lumbar spinal fusion surgery (86.8%). The mean duration of pain experience prior to surgery was 2.93 years.

### Baseline Comparison of Demographic and Disease Characteristics Between Groups

Following univariate analysis to determine baseline differences, it was observed that the demographic data for subjects in the PCA group were mostly comparable to those in the parecoxib group. However, several statistically significant differences in relation to baseline disease characteristics between them were found. Subjects in the parecoxib group were significantly more likely to experience pre-intervention higher levels of NRS scores and lower Barthel Index score than those receiving PCA treatment (both *p* < 0.01) (Table 1).

**Table 1:** Demographic and clinical characteristics of study participants by group.

**Variables**	**All participants (*****N*** **=** **68)**		**PCA (*****n*** **=** **36)**		**Parecoxib (*****n*** **=** **32)**		***P***
	***N***	**%**	***N***	**%**	***N***	**%**	
**Demographic data**							
**Sex**							0.86
Male	22	32.4	12	33.3	10	31.3	
Female	46	67.6	24	66.7	22	68.7	
**Monthly income**							0.61
Low (≤ 40,000)	36	52.9	18	50.0	18	56.2	
High (>40,000)	32	47.1	18	50.0	14	43.8	
**Alcohol**							0.11
No	60	88.2	29	80.6	31	96.9	
Ever use	8	11.8	7	19.4	1	3.1	
**Education**							0.55
Low (≤ 9 years)	45	66.2	25	69.4	20	62.5	
High (>9 years)	23	33.8	11	30.6	12	37.5	
**Job**							0.17
Employed	48	70.6	28	77.8	20	62.5	
Unemployed	20	29.4	8	22.2	12	37.5	
Age (years) (mean ± SD)	60.66	7.86	59.86	7.66	61.56	8.01	0.38
**Clinical characteristics**							
**Hypertension**							0.52
No	41	60.3	23	63.9	18	56.2	
Yes	27	39.7	13	36.1	14	43.8	
**Diabetes**							0.94
No	55	80.9	29	80.6	26	81.3	
Yes	13	19.1	7	19.4	6	18.7	
**Hepatitis**							0.74
No	63	92.6	33	91.7	30	93.7	
Yes	5	7.4	3	8.3	2	6.3	
**Peptic ulcer disease**							0.12
No	62	91.2	31	86.1	31	96.9	
Yes	6	8.8	5	13.9	1	3.1	
**Heart diseases**							0.10
No	65	95.6	33	91.7	32	100.0	
Yes	3	4.4	3	8.3	0	0	
**Ever use of analgesics before surgery**							0.87
No	9	13.2	5	13.9	4	12.5	
Yes	59	86.8	31	86.1	28	87.5	
Initial NRS (mean ± SD)	5.91	1.49	5.06	1.16	6.67	1.35	<0.01
Initial Barthel Index (mean ± SD)	33.7	7.95	37.7	8.42	30.1	5.54	<0.01
Disease duration of pain (mean ± SD)	2.93	4.33	3.14	4.74	2.69	3.87	0.70

### Primary Outcomes Between the Two Groups

**Figure 1:**
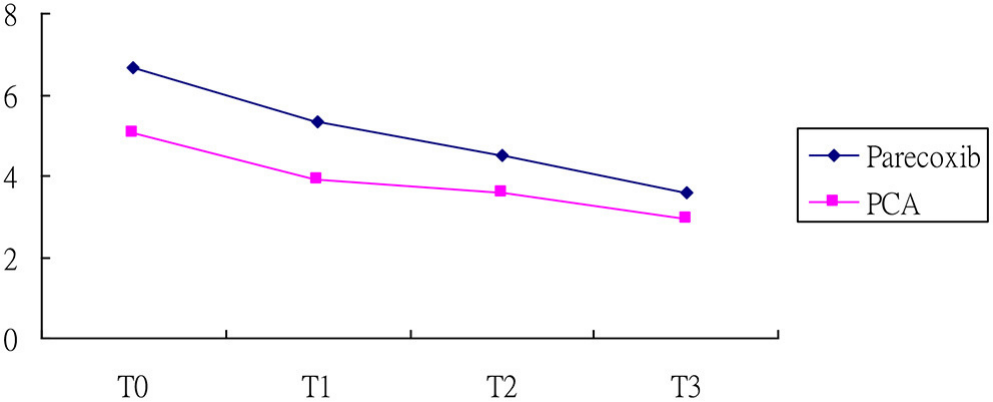
NRS score of patients in the parecoxib and PCA. T0, at 12 h after surgery; T1, at 24 h after surgery; T2, at 48 h after surgery, and T3, at 72 h after surgery.

Table 2 displays the GEEs results of NRS scores for the two groups. A notable difference was demonstrated for baseline NRS scores between them. Additionally, the NRS scores at T2 and T3 were statistically different from those at T0, implying a maturation effect may occur. After adjusting for the maturation effect together with baseline heterogeneity by GEEs model, the reduction slope of NRS score was evident for the parecoxib group compared with the PCA group, particularly in the third time measurement that reached statistically significance (*p* < 0.01; Figure 1). In addition, no rescue analgesia was required during the perioperative period.

**Table 2:** GEEs model of the NRS scores among subjects (*n* = 68).

**Variable**	**Regression coefficient**	**Standard error**	***P***
Intercept	5.06	0.36	<0.01
Group 1 (parecoxib) vs. Group 0 (PCA)	1.61	0.33	<0.01
T1 vs. T0	−1.16	0.13	<0.01
T2 vs. T0	−1.47	0.15	<0.01
T3 vs. T0	−2.13	0.17	<0.01
Interaction of T1 and group	−0.18	0.22	0.41
Interaction of T2 and group	−0.58	0.28	0.09
Interaction of T3 and group	−0.94	0.34	<0.01

**Figure 2:**
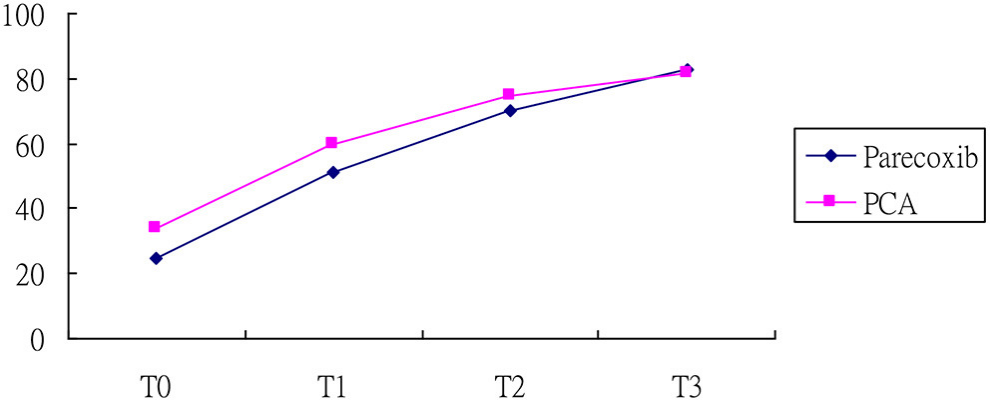
Barthel index of patients in the parecoxib and PCA. T0, at 12 h after surgery; T1, at 24 h after surgery; T2, at 48 h after surgery, and T3, at 72 h after surgery.

Regarding the mobility change scores between the two groups, the analysis revealed that the Barthel Index score increased with time, which supported the occurrence of a maturation effect (*p* < 0.01). The subjects in the parecoxib group reported lower Barthel Index scores than those in the PCA group at baseline (*p* < 0.01) (Table 3). After controlling for baseline differences and maturation effect, the increased level of Barthel Index in the parecoxib group was significantly higher than that in PCA group at both T2 and T3, with β values of 4.36 and 6.26 (all *p* values < 0.05; Figure 2), respectively.

**Table 3:** GEEs model of the Barthel index scores among subjects (*n* = 68).

**Variable**	**Regression coefficient**	**Standard error**	***P***
Intercept	33.82	2.49	<0.01
Group 1 (parecoxib) vs. Group 0 (PCA)	−8.85	2.04	<0.01
T1 vs. T0	25.94	1.49	<0.01
T2 vs. T0	40.78	1.48	<0.01
T3 vs. T0	47.91	1.50	<0.01
Interaction of T1 and group	0.04	2.62	0.99
Interaction of T2 and group	4.36	2.07	0.04
Interaction of T3 and group	6.26	1.93	<0.01

## Discussion

For those with degenerative lumbar disease, lumbar spinal fusion surgery has become an increasingly common therapeutic option. A persistent challenge in a significant number of cases has been postoperative pain, leading to delay of early ambulation after surgery, thereby resulting in delayed recovery and increased risk of mortality (4, 5). Currently, various preemptive analgesic strategies have been developed to address the postoperative pain issue, such as treatment with PCA or parecoxib. However, the studies designed to directly compare the effects of these two methods have been somewhat limited and sometimes controversial (11–13). Most importantly, previous researchers, periodically, neglected to account for specific baseline differences between groups and for autocorrelation among subjects across time, which could have prejudiced conclusions. The present study is possibly the first study to compare the impacts on pain and daily activities by PCA and parecoxib using the GEEs model.

After adjusting for baseline differences between the two groups and for maturation effects, as determined by the GEEs model, it was observed that the perioperative administration of parecoxib was related to a higher level of postoperative daily activities, when compared to the PCA group. However, since no previous studies have been conducted comparing the impact of PCA and parecoxib, on daily activities no direct comparison of results is therefore possible. Notwithstanding, the positive effect of parecoxib use on physical activity observed in this study was consistent with findings of earlier reports, and adds to the growing body of literature on this topic (10, 21). It is well known that morphine, or other opioids, is the most widely used intravenous drug for PCA, even as these common medications may be related to several adverse effects, such as nausea, vomiting, respiratory depression, sedation, constipation, confusion and urinary retention (8, 9). Taken the lung function as a example, it was found to be inversely associated with functional limitation. An earlier study showed that ~40% of patients with chronic lung diseases were prone to suffer some degree of disability and 68% lost at least one relevant function of daily living (22). Gao and colleagues also reported that those with poor lung function, as assessed by the FVC < 0.76 criterion, would be at nearly twice the risk of developing functional limitation (23).

In the present study, those receiving parecoxib had a significantly greater reduction in pain score than those receiving PCA after adjusting for the baseline heterogeneity along with inherent mature effect, and this beneficial effect was maintained for 72 h after undergoing surgery, echoing the earlier study finding (24). Several studies found that by suppressing the production of the pro-inflammatory cytokines containing TNF-alpha and IL-1beta, the COX-2 selective inhibitors were found to offer a number of advantages over classical non-steroidal anti-in?ammatory drugs, when used to treat postoperative pain (25, 26). These included an elimination of inhibitory effect on platelet function, thereby reducing the risk of blood loss. In addition, the use of COX-2 inhibitors less inflected impairment of bone healing (27), which may explain why the use of parecoxib could relieve pain to exhibit an opioid-sparing effect following lumbar spine fusion surgery.

In a head-to-head comparison of the effects of parecoxib and PCA, regarding pain and daily activities, the GEEs model was used to adjust for baseline differences and temporal maturation effect, allowing for a more robust determination of the study findings. Several limitations, however, should be noted when interpreting the results. First, all participants were drawn from a single hospital in southern Taiwan, so inferences drawn from the results may not be generalizable to populations in other geographic regions. However, prior to the implementation of the study, the sample size required to ensure statistical power was calculated and the sample size used in this study may, therefore, be considered satisfactory for the data analysis undertaken. Previous studies also faced barriers to their generalizability, because of participants' restricted ethnicity, geographic location, nationality, and the nature of the medical data available, suggesting that this limitation is not unique to our study. Second, the application of an observational non-randomized study may have weakened the internal validity of the research. This is because the current study was not randomized and there may have been disparities between two groups in the surgerical procedures that were not measured to possibly affect the findings herein. Nevertheless, in this investigation, we employed the GEEs model to control for baseline differences between the two groups, and further considered the potential maturation effects; this approach differed from the priori conventional repeated measures and would likely reduce the probability of inflated type I error to some extent (28). Finally, the future prospective randomized trials upon the larger sample size are needed to overcome the limitations of this study to provide more definite evidence of the findings reported.

## Conclusion

As far as the authors are aware, this is the first study to directly compare the different effects on pain and activities of daily living of the infusion of parecoxib and PCA, from a longitudinal perspective. After using the repeated measure of GEEs methodology, the results demonstrated that use of an intravenous infusion pump for parecoxib provided superior analgesic efficacy after lumbar spinal fusion surgery, when compared to PCA. As compared to use of PCA, using the parecoxib pump remarkably reduced the NRS scores throughout the 72-h study period, and patients who receive this treatment also reported higher levels of physical activity. This research provides evidence of the positive effects of Parecoxib use among those with lumbar spinal fusion surgery, particularly in terms of reducing postsurgical pain and facilitating movement.

## Data Availability Statement

The raw data supporting the conclusions of this article will be made available by the authors, without undue reservation.

## Ethics Statement

The studies involving human participants were reviewed and approved by Institutional Review Board and Ethics Committee of Buddhist Dalin Tzu Chi Hospital. The patients/participants provided their written informed consent to participate in this study.

## Author Contributions

S-CC, J-CC, T-IC, and T-YT: study concept and design. S-CC, C-MC, and T-YT: acquisition of data. S-CC and T-YT: data analysis. J-CC and HH: project management. S-CC, HL, HH, T-IC, and T-YT: writing. All authors gave final approval of the version to be published and agreed to be accountable for all aspects of the work.

## Conflict of Interest

The authors declare that the research was conducted in the absence of any commercial or financial relationships that could be construed as a potential conflict of interest.

## Publisher's Note

All claims expressed in this article are solely those of the authors and do not necessarily represent those of their affiliated organizations, or those of the publisher, the editors and the reviewers. Any product that may be evaluated in this article, or claim that may be made by its manufacturer, is not guaranteed or endorsed by the publisher.
